# The three cytokines IL-1β, IL-18, and IL-1α share related but distinct secretory routes

**DOI:** 10.1074/jbc.RA119.008009

**Published:** 2019-04-02

**Authors:** Victor S. Tapia, Michael J. D. Daniels, Pablo Palazón-Riquelme, Matthew Dewhurst, Nadia M. Luheshi, Jack Rivers-Auty, Jack Green, Elena Redondo-Castro, Philipp Kaldis, Gloria Lopez-Castejon, David Brough

**Affiliations:** From the ‡ Lydia Becker Institute of Immunology and Inflammation, University of Manchester, Manchester M13 9PT, United Kingdom; the § Division of Neuroscience and Experimental Psychology, School of Biological Sciences, Faculty of Biology, Medicine and Health, Manchester Academic Health Science Centre, University of Manchester, AV Hill Building, Oxford Road, Manchester M13 9PT, United Kingdom,; the ¶ UK Dementia Research Institute, University of Edinburgh, College of Medicine and Veterinary Medicine, 49 Little France Crescent, Edinburgh EH16 4SB, Scotland, United Kingdom,; the ‖ Division of Infection, Immunity, and Respiratory Medicine, School of Biological Sciences, Faculty of Biology, Medicine and Health, Manchester Collaborative Centre of Inflammation Research, Manchester Academic Health Science Centre, Core Technology Facility, University of Manchester, Manchester M13 9PT, United Kingdom,; the ** International Centre for Infectiology Research, INSERM U1111, CNRS UMR5308, École Normale Supérieure de Lyon, Claude Bernard Lyon 1 University, 69100 Lyon, France,; §§ MedImmune Ltd., Aaron Klug Building, Granta Park, Cambridge CB21 6GH, United Kingdom, and; the ‡‡ Institute of Molecular and Cell Biology (IMCB), A*STAR (Agency for Science, Technology and Research), Department of Biochemistry, National University of Singapore (NUS), Singapore 119007, Singapore

**Keywords:** inflammasome, inflammation, interleukin 1 (IL-1), NLRP3, caspase 1 (CASP1), calpain, cytokine, immunology, immune cell, neutrophil

## Abstract

Interleukin (IL)-1 family cytokines potently regulate inflammation, with the majority of the IL-1 family proteins being secreted from immune cells via unconventional pathways. In many cases, secretion of IL-1 cytokines appears to be closely coupled to cell death, yet the secretory mechanisms involved remain poorly understood. Here, we studied the secretion of the three best-characterized members of the IL-1 superfamily, IL-1α, IL-1β, and IL-18, in a range of conditions and cell types, including murine bone marrow–derived and peritoneal macrophages, human monocyte–derived macrophages, HeLa cells, and mouse embryonic fibroblasts. We discovered that IL-1β and IL-18 share a common secretory pathway that depends upon membrane permeability and can operate in the absence of complete cell lysis and cell death. We also found that the pathway regulating the trafficking of IL-1α is distinct from the pathway regulating IL-1β and IL-18. Although the release of IL-1α could also be dissociated from cell death, it was independent of the effects of the membrane-stabilizing agent punicalagin, which inhibited both IL-1β and IL-18 release. These results reveal that in addition to their role as danger signals released from dead cells, IL-1 family cytokines can be secreted in the absence of cell death. We propose that models used in the study of IL-1 release should be considered context-dependently.

## Introduction

Understanding mechanisms of unconventional protein secretion is a fundamental question of cell biology. Its importance is underscored by the biomedical relevance of many unconventionally secreted proteins. This is typified by the unconventionally secreted members of the interleukin (IL)-1^8^ cytokine family that have established roles in host-defense responses and in inflammatory responses that contribute to disease (1). The ancestral IL-1 family consists of IL-1β, IL-1α, IL-1Ra, IL-36Ra, IL-36α, IL-36β, IL-36γ, IL-37, and IL-38, with IL-18 and IL-33 having a distinct ancestry, but based on structural homology, receptor binding and immunomodulatory function remain part of the IL-1 superfamily (2). The best-characterized members of the IL-1 family are IL-1α, IL-1β, and IL-18, and all are released via unconventional pathways.

IL-1β, IL-1α, and IL-18 are initially produced as precursor pro-forms. In cells of hematopoietic lineage, such as macrophages, expression of precursor forms of IL-1β and IL-1α occurs after stimulation of membrane pattern recognition receptors such as TLR4, which can be activated by bacterial endotoxin for example, whereas pro-IL-18 is constitutively expressed. Both precursor forms of IL-1β and IL-18 are cleaved directly by the protease caspase-1, which then also, indirectly, influences Ca^2+^- and calpain-dependent processing of pro-IL-1α (3). Activation of caspase-1 occurs after the assembly of macromolecular protein complexes called inflammasomes, upon which caspase-1 is activated. Inflammasomes are formed by a cytosolic pattern recognition receptor, the best-studied of which is NLRP3, which nucleates oligomerization of an adaptor protein, ASC, into an inflammasome complex (4). A consequence of inflammasome activation is an inflammatory form of cell death called pyroptosis (5). Thus, a consequence of studying IL-1 release after inflammasome activation has been the concomitant death of the secreting cell, so it has long been considered that IL-1β release occurred through membrane rupture and lysis (6, 7). However, there are numerous examples, namely in human monocytes and neutrophils, where inflammasome activation can drive the release of IL-1β in the absence of cell death (8, 9). We and others have reported that the mechanism of secretion of IL-1β may depend on an alteration in membrane permeability (10, 11). Furthermore, the recent discovery that caspase-1 also cleaves gasdermin D, which subsequently forms pores in the plasma membrane that could allow passage of IL-1β (12, 13), and that a polybasic motif in the mature IL-1β domain could target it to phosphatidylinositol 4,5-bisphosphate–rich domains in the plasma membrane (14) have established that the mechanism of secretion is more complicated than simply membrane rupture. The mechanism through which IL-18 and IL-1α are secreted and the question of whether they are common with IL-1β remain underexplored. Here, we show that IL-1β, IL-18, and IL-1α can be secreted when cell lysis is prevented and that IL-1β and IL-18 share a common mechanism that relies on gasdermin D–dependent plasma membrane permeabilization.

## Results

### Secretion of IL-1β depends on membrane permeability

**Figure 1.**
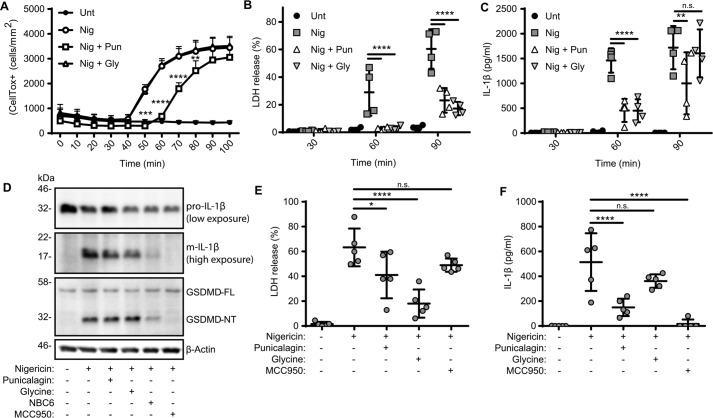
**IL-1β release after inflammasome activation depends on plasma membrane permeability.** *A–D*, LPS-primed (1 μg/ml, 2 h) iBMDMs were incubated with vehicle, punicalagin (*Pun*; 50 μm), or glycine (*Gly*; 5 mm) 15 min prior to activation with nigericin (*Nig*; 10 μm). *A*, membrane permeability was measured in real time by CellTox Green uptake (*n* = 4). Supernatants were assayed for cell death (*B*), measured as LDH release and normalized to a total cell lysis control, and for IL-1β release by ELISA (*n* = 4) (*C*). *D*, 1 h after nigericin stimulation, combined supernatant and cell lysate were analyzed for pro-IL-1β (31 kDa), m-IL-1β (17 kDa), gasdermin D (GSDMD) full-length (*FL*; 53 kDa), GSDMD N-terminal fragment (*NT*; 31 kDa), and β-actin (42 kDa) by Western blotting. *E* and *F*, LPS-primed (1 μg/ml, 3 h) mixed glia cultures were incubated with vehicle, punicalagin (50 μm), glycine (5 mm), or MCC950 (10 μm) 15 min prior to activation with nigericin (10 μm, 1 h). Supernatants were assayed for cell death, measured as LDH release (*E*), and IL-1β release by ELISA (*n* = 4) (*F*). *, *p* < 0.05; **, *p* < 0.01; ***, *p* < 0.001; ****, *p* < 0.0001; *n.s.*, nonsignificant, determined by two-way ANOVA with Sidak's post hoc analysis and compared with the nigericin-treated group (*A–C*) or one-way ANOVA with Dunnet's post hoc analysis compared with the nigericin-treated group (*E* and *F*). Western blots are representative of three independent experiments. *Error bars*, S.D.

Here we set out to test the initial hypothesis that release of IL-1β following NLRP3 inflammasome activation depended on a change in membrane permeability and not cell lysis. To interrogate this, we used the membrane-stabilizing reagent punicalagin. Punicalagin is a complex polyphenolic compound isolated from pomegranate extract that we previously reported to inhibit ATP-induced IL-1β release and uptake of the dye Yo-PRO-1 (as a measure of membrane permeability) with comparable potency and kinetics. Punicalagin also inhibits Yo-PRO-1 uptake and lactate dehydrogenase (LDH) release in response to membrane detergents digitonin and Triton X-100. Punicalagin also inhibits release of IL-1β independent of the inflammasome (10), strongly suggesting that under these conditions, it is acting at the plasma membrane. However, punicalagin is also reported to have additional effects, including potent anti-oxidant activity in macrophages (15), so some care must be taken when interpreting its effects. We also used the cytoprotectant glycine, which does not inhibit IL-1β release but limits cell lysis (16). We initially tested the effects of punicalagin (50 μm) and glycine (5 mm) directly on membrane permeability. LPS-primed (1 μg/ml, 2 h) immortalized mouse bone marrow–derived macrophages (iBMDMs) were incubated with CellTox Green dye, which would label cells after permeabilization, and were then incubated with vehicle, the NLRP3-activating stimulus nigericin (10 μm), or nigericin and either punicalagin or glycine, with the effects on dye uptake monitored by microscopy (for 100 min). In untreated cells, there was no dye uptake, whereas nigericin treatment caused a robust increase in fluorescence (Fig. 1*A*). Glycine had no effect on nigericin-induced dye uptake, whereas punicalagin significantly delayed it, suggesting that these two reagents were having significantly different effects on the plasma membrane (Fig. 1*A*). Sixty minutes of nigericin treatment of LPS-primed iBMDMs caused significant cell lysis, as measured by release of the cytoplasmic protein LDH (Fig. 1*B*). Nigericin-induced LDH release at 60 min was completely inhibited by both punicalagin and glycine. At 90 min of nigericin treatment, there was more LDH released, and this was still significantly decreased by punicalagin and glycine (Fig. 1*B*), suggesting that both reagents had a protective effect.

We next assessed the effects of punicalagin and glycine on nigericin-induced IL-1β release. Sixty minutes of nigericin treatment caused significant release of IL-1β that was partially inhibited by both punicalagin and glycine (Fig. 1*C*). At 90 min of nigericin treatment, punicalagin still decreased IL-1β release to some degree, but the inhibitory effect of glycine observed at 60 min was absent (Fig. 1*C*). These data suggest that whereas punicalagin was an effective inhibitor of IL-1β release, glycine slowed its release, likely by preventing release due to cell lysis. We then tested the effects of punicalagin and glycine on inflammasome-dependent processing of pro-IL-1β and gasdermin D by Western blotting of combined cell lysates and supernatants after nigericin treatment. Neither punicalagin nor glycine blocked the caspase-1–dependent processing of pro-IL-1β or gasdermin D, suggesting that they did not inhibit the inflammasome (Fig. 1*D*). Incubation with the NLRP3 inflammasome inhibitor NBC6 (17) or MCC950 (18) inhibited pro-IL-1β and gasdermin D processing as would be expected (Fig. 1*D*).

The iBMDM cells are useful for measuring inflammasome responses in general. However, it is important to determine whether the same mechanisms occur in cells that may experience tissue-specific disease conditions. Recent research has demonstrated that microglia form inflammasomes during Alzheimer's disease (19), and inflammasomes are implicated in the progression of a number of other neurological and neurodegenerative conditions (20). Thus, we investigated the relationship between IL-1β release and membrane permeability and cell death in cultures of glial cells (astrocytes and microglia) isolated from the brains of mice. These mixed glial cultures from the brains of neonates have microglial inflammasome responses very similar to those from primary microglia isolated from adult mice (21). LPS-primed (1 μg/ml, 3 h) glial cells were treated with the NLRP3 inflammasome activator nigericin (10 μm, 1 h), which caused robust cell death, and this was significantly reduced by punicalagin and by glycine (Fig. 1*E*). Nigericin also induced release of IL-1β from glial cells, which was inhibited by punicalagin (50 μm) but not by glycine (5 mm) (Fig. 1*F*). These data confirm the data obtained using the iBMDM cultures, which suggested that the release of IL-1β depends on a change in membrane permeability rather than cell lysis. These data also suggest that mechanisms of inflammasome activation and IL-1β release under these conditions are common between macrophages and microglia.

**Figure 2.**
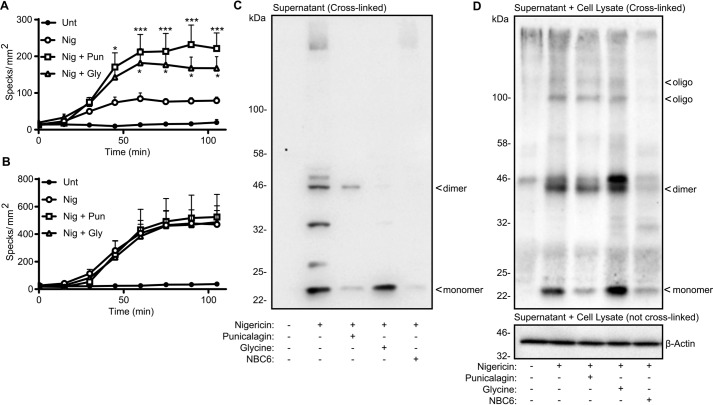
**ASC speck release depends on cell lysis.** *A* and *B*, LPS-primed (1 μg/ml, 2 h) ASC-mCherry iBMDMs were incubated with vehicle, punicalagin (*Pun*; 50 μm), or glycine (*Gly*; 5 mm) 15 min prior to activation with nigericin (*Nig*; 10 μm), and ASC speck formation was measured in real time without (*A*) or with (*B*) incubation of Z-VAD (50 μm) (*n* = 4). *C* and *D*, WT iBMDMs were treated as in *A* and *B* and activated with nigericin for 1 h. Supernatants (*C*) or combined supernatant and lysate (*D*) were cross-linked and analyzed for ASC or β-actin by Western blotting. ASC monomers (22 kDa), dimers (44 kDa), and oligomers are indicated. *, *p* < 0.05; ***, *p* < 0.001, determined by two-way ANOVA with Sidak's post hoc analysis and compared with the nigericin-treated group. Western blots are representative of three independent experiments. *Error bars*, S.D.

Activation of the NLRP3 inflammasome often forms an ASC speck, which itself can be released and is known to have pro-inflammatory effects in the extracellular space (22, 23). Using iBMDMs stably expressing ASC-mCherry (24), we observed that the number of visible ASC specks increased with nigericin treatment (10 μm) and that the number of visible specks increased further when the cells were treated with nigericin plus punicalagin (50 μm) or glycine (5 mm) (Fig. 2*A*). We then repeated this experiment, except we included the pan-caspase inhibitor Z-VAD, which allowed ASC speck formation, but inhibited potential ASC speck loss through caspase-1–dependent pyroptotic cell death. Incubation of the cells with Z-VAD increased the number of specks produced by nigericin treatment but had no further effect on the increase of ASC specks observed in nigericin-treated cells in the presence of punicalagin or glycine (Fig. 2*B*). These data suggest that ASC specks are released by pyroptotic cell lysis and that both punicalagin and glycine inhibited this release. This further supports a regulated mechanism for IL-1β release dependent upon a change in membrane permeability, as its release was unaffected by glycine (Fig. 1). We investigated the release of ASC further by analyzing the oligomeric forms released into the cell supernatant using cross-linking and Western blotting. LPS-treated iBMDMs did not release any ASC into the supernatant (Fig. 2*C*). Nigericin treatment caused the release of a monomeric and a range of oligomeric ASC forms, which was reduced by punicalagin treatment (Fig. 2*C*). Glycine treatment inhibited the release of high-molecular weight oligomeric ASC, but lower-molecular weight forms were released (Fig. 2*C*). This suggested that monomeric ASC may follow the same pathway out of the cell as IL-1β. The inflammasome inhibitor NBC6 inhibited the release of all forms of ASC, confirming that its release was inflammasome-dependent (Fig. 2*C*). These data suggest that oligomeric ASC and ASC specks are released by pyroptotic cell lysis but that changes in membrane permeability allow the release of monomeric ASC. Western blotting of combined cell lysates and supernatants after nigericin treatment confirmed this, showing that punicalagin and glycine did not inhibit ASC oligomerization in response to nigericin, whereas the inflammasome inhibitor NBC6 did (Fig. 2*D*).

### IL-18 and IL-1β follow a similar secretory route

**Figure 3.**
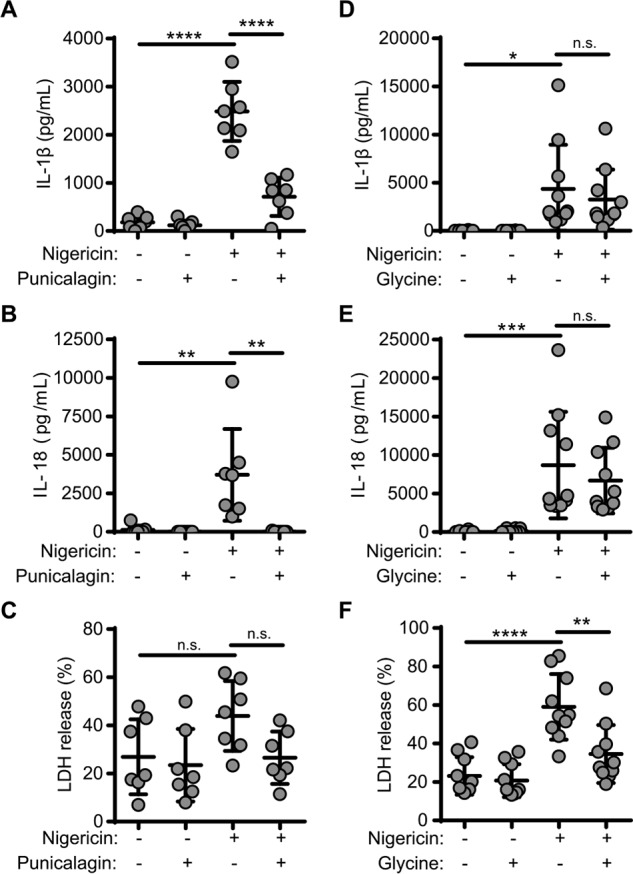
**IL-18 release during inflammasome activation depends on plasma membrane permeability.** *A–C*, LPS-primed (1 μg/ml, 2 h) MDMs were incubated with vehicle or punicalagin (25 μm) 15 min prior to activation with nigericin (10 μm, 45 min). Supernatants were assayed for IL-1β (*A*) and IL-18 (*B*) by ELISA (*n* = 8–9). *C*, cell death was measured as LDH release normalized to a total cell lysis control. *D–F*, LPS-primed (1 μg/ml, 2 h) MDMs were incubated with vehicle or glycine (5 mm) 15 min prior to activation with nigericin (10 μm, 45 min). Supernatants were assayed for IL-1β (*D*) and IL-18 (*E*) by ELISA (*n* = 8–9). *F*, cell death was measured as LDH release. *, *p* < 0.05; **, *p* < 0.01; ***, *p* < 0.001; ****, *p* < 0.001; *n.s.*, nonsignificant, determined by one-way ANOVA with Dunnet's post hoc analysis and compared with the nigericin-treated group. *Error bars*, S.D.

IL-18 is also produced as a precursor pro-form and is directly regulated by the inflammasome and caspase-1. However, the mechanisms underpinning release of IL-18 are poorly understood. In these studies, we used primary human monocyte–derived macrophage cultures (MDMs), which also allowed us to interrogate the release pathway in human cells. MDMs were primed with LPS (1 μg/ml, 4 h) and then stimulated with nigericin (10 μm, 45 min) to induce release of IL-1β and IL-18. Under these conditions, nigericin induced release of both IL-1β and IL-18, and in both cases release was inhibited by a 15-min pre-incubation with punicalagin (25 μm) (Fig. 3, *A* and *B*). Under these conditions, punicalagin did not significantly reduce nigericin-induced LDH release (Fig. 3*C*). Under the same conditions, incubation with glycine (5 mm) did not inhibit nigericin-induced IL-1β or IL-18 release from MDMs (Fig. 3, *D* and *E*) but did inhibit nigericin-induced LDH release (Fig. 3*F*), suggesting that the release of IL-18 was also dependent upon a change in membrane permeability. These data also suggest that IL-1β release after NLRP3 inflammasome activation is common in mouse and human macrophages, highlighting that under these conditions the mouse BMDMs are representative of a range of macrophage IL-1 release models.

### IL-1α is secreted via an alternative pathway

**Figure 4.**
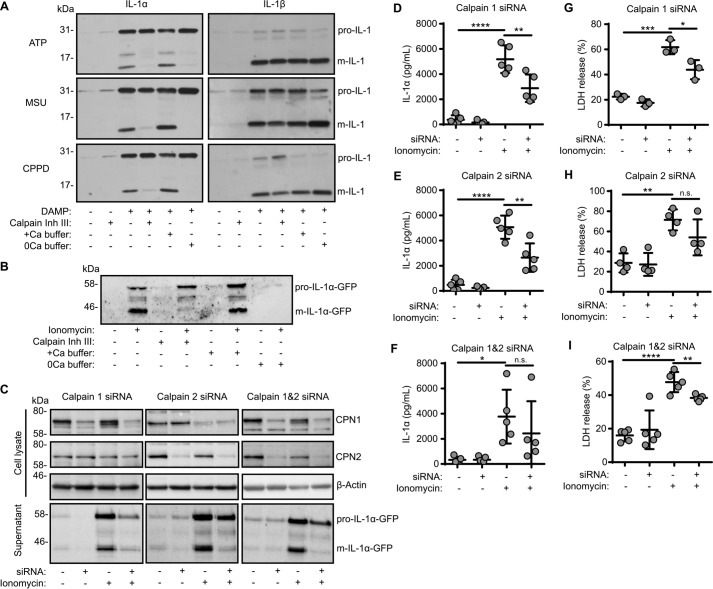
**IL-1α processing and release depends on calpains 1 and 2.** *A*, LPS-primed (1 μg/ml, 2 h) peritoneal macrophages were incubated with calpain inhibitor III (50 μm) or in calcium-containing (+*Ca*) or calcium-free (*0Ca*) buffers 15 min prior to activation with ATP (5 mm, 1 h), MSU (250 μg/ml, 1 h), or CPPD (250 μg/ml, 1 h). Supernatants were analyzed for IL-1α (pro-form, 31 kDa; mature form, 17 kDa) and IL-1β (pro-form, 31 kDa; mature form, 17 kDa) by Western blotting. *B*, HeLa cells were transfected with pro-IL-1α-GFP (24 h) and then incubated with calpain inhibitor III (40 μm) or in calcium-containing (+*Ca*) or calcium-free (*0Ca*) buffers 15 min prior to activation with ionomycin (10 μm, 1 h). Supernatants were analyzed for IL-1α-GFP (pro-form, 58 kDa; mature form, 44 kDa) by Western blotting. *C–I*, HeLa cells were transfected with calpain 1, calpain 2, or scrambled siRNA (48 h), transfected with pro-IL-1α-GFP (24 h), and treated as in *B. C*, cell lysates were analyzed for calpain 1 (*CPN1*; 75 kDa), calpain 2 (*CPN2*; 75 kDa), and β-actin (42 kDa) and supernatants for m-IL-1α-GFP (44 kDa) by Western blotting. Supernatants were assayed for IL-1α release measured by ELISA (*D–F*) and cell death, measured as LDH release (*n* = 4) (*G–I*). *, *p* < 0.05; **, *p* < 0.01; ***, *p* < 0.001; ****, *p* < 0.0001; *n.s.*, nonsignificant, determined by one-way ANOVA with Dunnet's post hoc analysis compared with the ionomycin-treated group. Western blots are representative of at least three independent experiments.

Release of IL-1α can also be regulated by inflammasomes, albeit indirectly (3). Release of IL-1α is thought to be dependent upon Ca^2+^-activated calpain proteases (3), although a number of other proteases are known to also cleave pro-IL-1α (25). In primary mouse peritoneal macrophages primed with LPS (1 μg/ml, 2 h) and treated with NLRP3 inflammasome activator ATP (30 min) or MSU or CPPD crystals (250 μg/ml, 1 h), there was a release of both mature IL-1α and mature IL-1β into the culture supernatants (Fig. 4*A*). Incubation of the cells with calpain inhibitor III (50 μm) or in Ca^2+^-free buffer with subsequent NLRP3 inflammasome activation by ATP or by MSU or CPPD crystals inhibited the release of mature IL-1α but not IL-1β (Fig. 4*A*), highlighting the divergence of the secretory signaling pathways. Whereas the pharmacological data produced by us and others has strongly suggested that a calpain protease is required for pro-IL-1α processing, genetic proof for this is lacking. To address this, we generated a reconstituted cellular model of IL-1α secretion. Macrophage cells are difficult to transfect, and they respond to DNA with inflammasome activation. Thus, we investigated whether we could model IL-1α release in easy-to-transfect HeLa cells. HeLa cells were transfected to express pro-IL-1α-GFP and were then treated with the Ca^2+^ ionophore ionomycin (10 μm, 1 h) to induce calpain activation and IL-1α processing and release (Fig. 4*B*). In this model, release of mature IL-1α was also inhibited by calpain inhibitor III (40 μm) and by the removal of extracellular Ca^2+^, suggesting that the pathways of IL-1α processing and release in our reconstituted HeLa cell model are representative of the pathways of IL-1α release in primary macrophages (Fig. 4*B*). There are 14 members of the calpain family in mammals, with calpains 1 and 2 the best characterized (26). We therefore knocked down expression of calpain 1, calpain 2, or both calpains 1 and 2 in HeLa cells using siRNAs that were transfected to express pro-IL-1α-GFP and then treated them with ionomycin to induce calpain activation and mature IL-1α release (Fig. 4*C*). We were able to selectively knock down expression of calpain 1 and calpain 2 (Fig. 4*C*). Knockdown of either calpain inhibited release of mature IL-1α as determined by Western blotting (Fig. 4*C*), confirming the importance of calpain to this pathway and suggesting that both calpains 1 and 2 can process pro-IL-1α. This was further confirmed by ELISA of the HeLa cell supernatants after ionomycin treatment, which contained significantly less IL-1α after calpain knockdown (Fig. 4, *D–F*). Calpain knockdown also significantly reduced ionomycin-induced cell death (Fig. 4, *G–I*).

**Figure 5.**
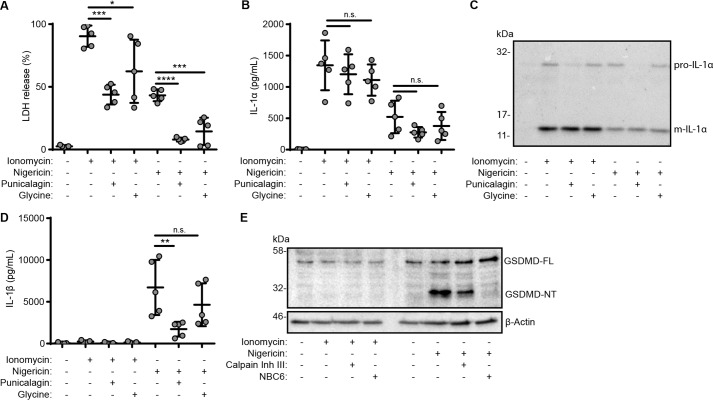
**IL-1α release can occur independently of cell lysis.** *A–D*, LPS-primed (1 μg/ml, 4 h) primary mouse BMDMs were incubated with vehicle, punicalagin (*Pun*; 50 μm), or glycine (*Gly*; 5 mm) 15 min prior to activation with ionomycin (10 μm, 1 h) or nigericin (10 μm, 1 h). *A*, cell death was measured as LDH release (*n* = 5). IL-1α release was assayed by ELISA (*n* = 5) (*B*) or analyzed for pro-IL-1α (31 kDa) and m-IL-1α (17 kDa) by Western blotting (*C*). *D*, IL-1β release was assayed by ELISA (*n* = 5). *E*, BMDMs were treated as previously but incubated with calpain inhibitor III (40 μm) or NBC6 (20 μm) prior to ionomycin or nigericin treatment. Combined supernatants and lysates were analyzed for GSDMD full-length (*FL*; 53 kDa), GSDMD N-terminal fragment (*NT*; 31 kDa), and β-actin (42 kDa) by Western blotting. *, *p* < 0.01; **, *p* < 0.001; ***, *p* < 0.0001; ****, *p* < 0.0001; *n.s.*, nonsignificant, determined by one-way ANOVA with Dunnet's post hoc analysis compared with the ionomycin- or nigericin-treated groups. Western blots are representative of two independent experiments. *Error bars*, S.E.

IL-1α may be released and processed as a consequence of cell death (27, 28). Whereas IL-1β–secreting cells often undergo a pyroptotic or pyronecrotic cell death (6, 29), there are examples where IL-1β is secreted in the absence of cell death (9, 30). Our data above suggest that IL-1β release depends upon membrane permeability, but not cell lysis *per se*. We therefore sought to determine whether Ca^2+^/calpain-dependent release of IL-1α requires cell lysis. We previously published that punicalagin does not inhibit IL-1α release, suggesting a separate pathway of secretion from IL-1β (10). To directly compare the effects of punicalagin and glycine on the release of IL-1α to IL-1β and IL-18, we treated primary mouse BMDMs with LPS (1 μg/ml, 4 h) and then incubated them with punicalagin (50 μm) or glycine (5 mm) and then stimulated with ionomycin (10 μm, 1 h) to activate calpain or with nigericin (10 μm, 1 h) to activate the NLRP3 inflammasome. Both punicalagin and glycine inhibited ionomycin and nigericin-induced cell death (Fig. 5*A*). However, neither punicalagin nor glycine inhibited release of IL-1α in response to ionomycin or nigericin (Fig. 5, *B* and *C*). Ionomycin did not cause release of IL-1β (Fig. 5*D*) or cleavage of gasdermin D (Fig. 5*E*), and nigericin-induced cleavage of gasdermin D was not prevented by calpain inhibition but was prevented by NLRP3 inflammasome inhibition with NBC6 (Fig. 5*E*). Together, these data suggest that, whereas in many cell types, close associations between the release of related cytokines IL-1α and IL-1β and cell death are observed, these processes can be dissociated from each other. Release of IL-1β appears to rely on a change in membrane permeability dependent upon gasdermin D cleavage (31, 32). Calpain-dependent IL-1α release appears to be independent of cell lysis but relies on alternative, but seemingly parallel, pathways to IL-1β.

**Figure 6.**
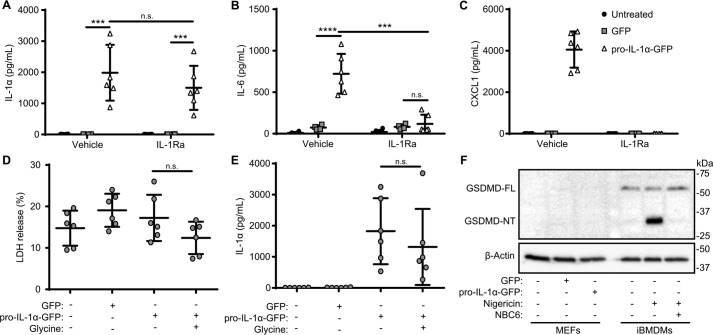
**IL-1α is released from viable MEF cells.** *A–C*, MEF cells were transfected with pro-IL-1α-GFP or with GFP alone (48 h) and treated with IL-1Ra (10 μg/ml, 48 h). Supernatants were assayed for IL-1α (*A*), IL-6 (*B*), and CXCL1 (*C*) release by ELISA (*n* = 6). *D* and *E*, MEF cells transfected as previously were treated with glycine (5 mm, 24 h). Supernatants were assayed for cell death, measured as LDH release (*D*), and IL-1α release measured by ELISA (*n* = 4) (*E*). *F*, cell lysates from MEFs, transfected as previously, and LPS-primed iBMDMs, incubated with NBC6 (20 μm) and stimulated with nigericin (10 μm, 1 h), were analyzed for GSDMD full-length (*FL*; 53 kDa), GSDMD N-terminal fragment (*NT*; 31 kDa), and β-actin (42 kDa) by Western blotting. ***, *p* < 0.0001; ****, *p* < 0.0001; *n.s.*, nonsignificant, determined by one-way ANOVA with Sidak's post hoc analysis with multiple comparisons (*A* and *B*), or one-way ANOVA with Dunnet's post hoc analysis compared with the pro-IL-1α-GFP–transfected group (*D* and *E*). *n.d.*, not detected. Western blots are representative of two independent experiments. *Error bars*, S.E.

The above data suggested that IL-1α could be secreted from cells independently of plasma membrane rupture. We next examined the release of IL-1α in an alternative cell model. Cellular senescence is a barrier to tumorigenesis in response to oncogenic stresses by forcing cells to permanently exit from the cell cycle (33). Although beneficial early in an organism's life, at older ages, cellular senescence can promote tissue disruption and can paradoxically be pro-tumorigenic by virtue of the senescence-associated secretory phenotype (SASP) (33). The SASP is the secretion of pro-inflammatory cytokines from senescent cells that can promote paracrine senescence (34). A defining feature of cellular senescence is the expression of IL-1α, which is critical for the development of the SASP and in some cases can drive senescence (34), whereas in other cases, blockade of IL-1α reduces the SASP, but the cell still senesces (35, 36). It is not currently understood whether IL-1α secretion, required for SASP development, results in death of the secreting cell. To address this, we used immortalized mouse embryonic fibroblast (MEF) cells (37), as cellular senescence is often studied with fibroblasts. To recapitulate a SASP-like phenotype, MEF cells were transfected to express pro-IL-1α-GFP. IL-1α was released from transfected MEF cells when assayed 48 h after transfection (Fig. 6*A*). IL-6 and CXCL1 levels were also significantly increased after 48 h in pro-IL-1α-GFP–transfected groups but not in GFP-transfected or nontransfected groups (Fig. 6, *B* and *C*). In addition, 48 h after treatment with the IL-1 receptor antagonist IL-1Ra (1 μg/ml added every 24 h for the duration of the experiment), CXCL1 and IL-6 secretion were significantly reduced, whereas levels of IL-1α were not affected (Fig. 6, *A–C*). These data suggest that IL-1α is secreted over time to induce the release of IL-6 and CXCL1. Importantly, there was no significant cell death over the duration of this experiment, and glycine (5 mm) added for the last 24 h of incubation had no effect on IL-1α release (Fig. 6, *D* and *E*). These data confirm the finding above that IL-1α can be released in the absence of cell lysis and through a separate pathway to IL-1β and IL-18 in a variety of cell types. The release of IL-1α by these MEF cells was independent of gasdermin D cleavage, as it was not expressed (Fig. 6*F*), again highlighting the difference between IL-1α and IL-1β release.

## Discussion

Members of the IL-1 family have been described as canonical DAMPs, and indeed there is evidence to support this (38). For an IL-1 family member to act like a canonical DAMP, it would be released as a result of cellular death or injury. However, there is also evidence to suggest that some IL-1 family members can be released in the absence of cell death and may therefore also act as actively secreted cytokines (39, 40). For example, there is evidence that caspase-1–dependent processing and secretion of IL-1β from macrophages and neutrophils can occur in the absence of cell lysis (9, 30), although from these studies, the measurements are from cell populations rather than single cells, which may mask any correlations with cell death and IL-1β release at the single-cell level (10). An often reported consequence of inflammasome activation in macrophages is cell death, and although release of IL-1β can be temporally separated from release of a lytic marker such as LDH, it seems that a complete loss of cell integrity is inevitable in many cases (29). However, cytoprotectants such as glycine can be used to prevent cell lysis after inflammasome activation in macrophages but still allow the release of mature IL-1β (16, 41).

The release of IL-1β from macrophages can also be blocked by the membrane-stabilizing agent punicalagin (10). Here, we showed that glycine, while blocking cell lysis and ASC speck/oligomer release, did not inhibit NLRP3-dependent release of IL-1β and that punicalagin was at least partially effective against both cell lysis and IL-1β release. Pyroptosis leads to release of active IL-1β and concomitant release of ASC specks capable of being taken up by other cells and propagating an inflammatory response (22, 23). This process of ASC speck release was recently implicated in models of Alzheimer's disease where ASC specks were shown to seed β-amyloid plaques (19). Here, we report conditions allowing release of IL-1β and monomeric ASC from cells with active inflammasomes, but where release of ASC oligomers and specks is blocked. By dissociating IL-1β secretion from ASC speck release, we have provided conditions that allow for novel insights to be made into the individual roles played by these inflammatory factors in future studies.

The IL-1β release inhibitor punicalagin influenced the permeability of the plasma membrane to the dye CellTox Green, suggesting that it is a specific change in membrane permeability rather than cell lysis *per se* that is allowing release of IL-1β. This was also the case for NLRP3-dependent IL-1β release in human macrophages and for the related cytokine IL-18, suggesting that they may share a common exit route from the cell. Identifying that, under the stated conditions, the pathway of IL-1β release is common between mouse and human macrophages and different subtypes of macrophage allows us to further reliably interpret and compare studies in different cell types and from different species. Although the secretions of IL-1α and IL-1β from macrophages in response to NLRP3 inflammasome–activating stimuli were previously suggested to follow a common secretory route based on kinetics and inhibitor sensitivity (42), our data suggest that in fact the secretory mechanisms are distinct. IL-1α and IL-1β are closely related molecules, with IL-1α arising as a result of a gene duplication event of IL-1β (2). Significant divergence between IL-1α and IL-1β has occurred since the duplication event at the amino acid level, particularly within the pro-domain, although there is very little evidence of divergence in mechanisms of secretion. Here, we provide evidence in macrophages that the secretion of IL-1α is independent of IL-1β and IL-18. We have also modeled the IL-1α release pathway in easy-to-transfect cell lines (HeLa and MEF), allowing us to further conclude that IL-1α may be actively secreted from cells, which may be important for development of the SASP and thus cellular senescence. This discovery opens further avenues of research where we can now address the other contexts in which IL-1α is actively secreted from living cells. Our studies in the MEF cells suggest that IL-1α secretion is independent of gasdermin D. It should be noted, however, that IL-1α release from BMDMs infected with a mutant strain of *Staphylococcus aureus* was less from gasdermin D KO cells compared with WT (32). Also, whereas it is now becoming well-accepted that release of IL-1β is gasdermin D–dependent, a delayed gasdermin D–independent mechanism of IL-1β release has also been described (14).

Overall, these data have broad implications and suggest that IL-1 family members behave both as DAMPs and as actively secreted cytokines. Our use of a senescence-like model to study IL-1α secretion highlights the value of using context-specific models when studying IL-1 release pathways. Cellular senescence, a process in which there is no overt cell death, now provides a context for the nonlytic release of IL-1α. Likewise, DAMP-dependent release of IL-1 from macrophages may not present us with a unifying pathway to describe IL-1β secretion in all circumstances, and we are learning that activations of canonical and alternative inflammasomes have very different effects on ASC speck formation and cell death (8, 43).

## Experimental procedures

### Antibodies and reagents

Antibodies used targeted mouse IL-1β (AF-401-NA, R&D Systems), mouse gasdermin D (ab209845, Abcam), ASC (AL177, Adipogen), mouse IL-1α (AF-400-NA, R&D Systems), human calpain 1 (ab39170, Abcam), human calpain 2 (ab39165, Abcam), and β-actin-HRP (A3854, Sigma). Pharmacological agents used were punicalagin (Sigma), glycine (Sigma), NBC6 (synthesized in house (17)), MCC950 (CP-456773, Sigma), Z-VAD-fluoromethyl ketone (Merck), calpain inhibitor III (Merck), nigericin (Sigma), adenosine triphosphate (Sigma), ionomycin (Sigma), monosodium urate crystals (InVivoGen), calcium pyrophosphate dihydrate crystals (InVivoGen), and IL-Ra (Kineret®, Amgen). All other materials were from Sigma-Aldrich unless specified.

### Cell culture

Mouse iBMDMs, obtained from Claire Bryant (Department of Veterinary Medicine, University of Cambridge), and ASC-mCherry iBMDMs (23) were cultured in Dulbecco's modified Eagle's medium (DMEM), 10% fetal bovine serum (FBS; Life Technologies), 100 units/ml penicillin, and 100 μg/ml penicillin-streptomycin (PenStrep). The iBMDMs were seeded overnight at a density of 0.75 × 10^6^ cells/ml. Murine mixed glia cultures were prepared from brains isolated from 3–4-day-old C57BL/6 mouse pups (Envigo). All animal procedures were performed with appropriate personal and project licenses in place, in accordance with the Home Office (Animals) Scientific Procedures Act (1986) and approved by the Home Office and the local Animal Ethical Review Group, University of Manchester. As described previously (20), brain tissue was mechanically digested, and cells were maintained in DMEM, 10% FBS, PenStrep until 80% confluence was reached (10–13 days *in vitro*). Cultures were then reseeded at a density of 1.7 × 10^5^ cells/ml. After a further 2–3 days, cells were ready for experimentation.

Murine peritoneal macrophages were isolated from C57BL/6 mice. Mice were anesthetized with isoflurane (induced at 3–4% in 33% O_2_, 67% NO_2_, maintained at 1–2%), and peritoneal cavities were lavaged with 6 ml of RPMI. Peritoneal macrophages were cultured in DMEM, 10% FBS, PenStrep and seeded overnight at a density of 1 × 10^6^ cells/ml before the experiment the following day.

Murine primary BMDMs were prepared by flushing femurs of C57BL/6 mice. Red blood cells were lysed with ACK lysis buffer (Lonza), and BMDMs were generated by culturing the resulting marrow cells in 70% DMEM (containing 10% FBS, PenStrep) and 30% L929 mouse fibroblast–conditioned medium for 7–10 days. Primary BMDMs were seeded overnight at a density of 1 × 10^6^ cells/ml before the experiment.

The MEF cell line was derived from primary MEFs and immortalized with retroviral introduction of shRNA against p53 (pSuperRetro-sh53) (37). Cells were cultured in DMEM, 10% FBS, PenStrep. Before experiments, MEFs were seeded overnight at a density of 5 × 10^4^ cells/ml.

Human MDMs were generated as described previously (17). Briefly, peripheral blood mononuclear cells (PBMCs) were obtained from leukocyte cones from healthy donors (Service Blood and Transplant, Manchester, UK) with full ethical approval from the Research Governance, Ethics, and Integrity Committee at the University of Manchester (reference no. 2018-2696-5711). After a density centrifugation step using a Ficoll gradient, the PBMC layer was carefully removed, and monocytes were obtained from the PBMCs by positive selection with CD14 magnetic MicroBeads and an LS column (Miltenyi) for 15 min. Monocytes were differentiated into macrophages for 6 days (5 × 10^5^ cells/ml) in RPMI (containing 10% FBS, PenStrep) and in the presence of 0.5 ng/ml M-CSF (Peprotech). At day 3, half of the medium was removed and replaced with fresh medium to foster proliferation.

Human HeLa cells were cultured in DMEM, 10% FBS, PenStrep. HeLa cells were cultured overnight after seeding at a density of 5 × 10^4^ cells/ml.

### NLRP3 and calpain activation protocol

Cells were seeded in 96- or 48-well plates and primed with LPS (1 μg/ml) for 2 h (iBMDMs, peritoneal macrophages), 3 h (mixed glia), or 4 h (primary BMDMs, MDMs). After priming, the medium was changed to FBS-free DMEM, calcium-free buffer, or calcium-containing buffer (44). Calcium-free buffer was composed of 121 mm NaCl, 5.4 mm KCl, 0.8 mm MgCl_2_, 6 mm NaHCO_3_, 25 mm HEPES, 5 mm glucose, and 5 mm EGTA. Calcium-containing buffer was composed of 121 mm NaCl, 5.4 mm KCl, 0.8 mm MgCl_2_, 1.8 mm CaCl_2_, 6 mm NaHCO_3_, 25 mm HEPES, and 5 mm glucose. Cells were then treated with punicalagin (25 or 50 μm), glycine (5 mm), NBC6 (20 μm), MCC950 (10 μm), or calpain inhibitor III (40 and 50 μm) for 15 min. Cells were then stimulated by adding the NLRP3-activating stimuli ATP (5 mm), nigericin (10 μm), MSU (250 μg/ml), CPPD (250 μg/ml), or the Ca^2+^ ionophore ionomycin (10 μm).

### Live-cell imaging

Real-time membrane permeabilization assays were performed using iBMDMs seeded in 96-well plates. Cells were primed with LPS (1 μg/ml, 2 h). After priming, cells were incubated with CellTox Green (Promega); treated with punicalagin (50 μm), glycine (5 mm), and Z-VAD (50 μm); and stimulated with nigericin (10 μm, 100 min). Real-time ASC speck assays were performed in the same conditions, using ASC-mCherry iBMDMs. Images were captured every 10 or 15 min using an IncuCyte ZOOM System (Essen Bioscience) with a ×20/0.61 S Plan Fluor objective. Excitation and emission wavelengths were 440–480 and 504–544 nm for permeability assays and 565–605 and 625–705 nm for speck assays.

### Cell death analysis

Cell death was measured by assessing LDH release into cell culture supernatants, using the CytoTox 96 Non-Radioactive Cytotoxicity Assay (Promega) according to the manufacturer's instructions. Samples were quantified by reading absorbance at 490 nm in a Synergy HT microplate reader (Biotek Instruments). LDH release was expressed as a percentage normalized to a total cell lysis control, subtracting the background signal from cell culture medium.

### Cytokine release analysis

IL-1α, IL-1β, CXCL1, and IL-6 measurements were made in cell supernatants by using Duoset ELISA kits (R&D Systems) following the manufacturer's instructions. Human IL-18 was measured using the eBioscience kit (IL-18, BMS267/2MST). Samples were quantified using corrected values of 450 and 570 nm, reading absorbance in a Synergy HT microplate reader (Biotek Instruments).

### Western blot analysis

Western blot analysis was performed on supernatants and lysates for IL-1β, gasdermin D, ASC, IL-18, IL-1α, calpain 1, calpain 2, and β-actin. Samples were run on 10% (calpain 1 and calpain 2), 12% (ASC and pro-IL-1IL-1α-GFP), or 15% (IL-1α, IL-1β, IL-18, and gasdermin D) SDS-polyacrylamide gels. Gels were transferred using a Trans-Blot® Turbo^TM^ Transfer System (Bio-Rad) at 25 V for 7 min before blocking with 2.5% BSA in PBS, 1% Tween 20 (PBST) for 1 h at room temperature. Membranes were washed and incubated (4 °C) overnight in primary antibody in PBST, 0.1% BSA. Following this, membranes were washed and incubated with horseradish peroxidase–conjugated secondary antibodies (Dako) in PBST, 0.1% BSA for 1 h at room temperature. Finally, membranes were washed and incubated in Amersham Biosciences ECL Western Blotting Detection Reagent (GE Life Sciences) before exposure using a G:BOX gel doc system (Syngene).

### ASC oligomerization assay

iBMDMs were seeded into 24-well plates. Cells were primed with LPS (1 μg/ml, 2 h) and then incubated with punicalagin (50 μm), glycine (5 mm), or NBC6 (20 μm) for 15 min and stimulated with nigericin (10 μm, 60 min). For total oligomerization, cells were directly lysed in the well by the addition of 1% Triton X-100. Cell lysates were separated into a Triton X-100–soluble fraction and insoluble fraction by centrifugation at 6800 × *g* for 20 min at 4 °C. The insoluble pellets were cross-linked with disuccinimidyl suberate (2 mm; Thermo Fisher Scientific) for 30 min. Cross-linked pellets were further spun down at 6800 × *g* for 20 min and eluted in Laemmli buffer for SDS-PAGE. For detection of released ASC oligomers, supernatants were collected and detached cells were removed, and the supernatants were then concentrated by centrifugal filtering (Amicon 10K centrifugal filters) according to the manufacturer's instructions. The concentrated supernatants were chemically cross-linked with disuccinimidyl suberate for 30 min at room temperature before Laemmli buffer was added in preparation for Western blotting.

### IL-1α release from HeLa cells

HeLa cells were seeded into 24-well plates. Cells were transfected with plasmids expressing pro-IL-1α-GFP or GFP-only control (0.5 μg/well, 24 h) using Lipofectamine 3000 according to the manufacturer's instructions. For RNAi studies, HeLa cells were transfected with siRNA for calpain 1, calpain 2, or scrambled control (Santa Cruz Biotechnology, Inc.; 40 nm, 72 h) using Lipofectamine 3000. On the day of the ionomycin stimulus, medium was changed to DMEM, calcium-free buffer, or calcium-containing buffer, and cells were incubated with or without calpain inhibitor III (40 μm, 15 min) before stimulation with ionomycin (10 μm, 1 h).

### SASP-like IL-1α release model

MEF cells were seeded into 24-well plates. Cells were transfected with plasmids for pro-IL-1α-GFP or GFP expression (0.5 μg of DNA/well, 48 h) using Lipofectamine 3000. Transfected groups were treated with and without IL-1Ra (1 μg/ml, added at 0 and 24 h) and with or without glycine (5 mm, 24 h).

### Statistics

Data are presented as scatter plots with the mean ± S.D. indicated. Statistical analysis was performed using GraphPad Prism version 7 software. Accepted levels of significance were as follows: *, *p* < 0.05; **, *p* < 0.01; ***, *p* < 0.001; ****, *p* < 0.0001. Data with comparisons against a vehicle control were analyzed using one-way ANOVA followed by Dunnet's post hoc analysis, or with multiple comparisons with Sidak's post hoc analysis. Time courses were compared against a vehicle control by two-way ANOVA with Sidak's post hoc analysis. In one-way ANOVA, equal variance was evaluated with the Brown–Forsythe test, and transformations were performed where necessary. Experimental replicates (*n*) were defined as experiments performed on different passages of immortal cell lines (iBMDMs, HeLa, or MEF cells) or individual animal/human donors (primary BDMDs or MDMs, respectively).

## Author contributions

V. S. T., M. J. D., P. P.-R., M. D., N. M. L., J. R.-A., J. G., and E. R.-C. data curation; V. S. T., M. J. D., P. P.-R., M. D., N. M. L., J. R.-A., J. G., and E. R.-C. formal analysis; V. S. T. and D. B. validation; V. S. T., M. J. D., P. P.-R., M. D., N. M. L., J. R.-A., J. G., E. R.-C., G. L.-C., and D. B. investigation; V. S. T. visualization; V. S. T. methodology; V. S. T., P. K., G. L.-C., and D. B. writing-original draft; J. R.-A., E. R.-C., P. K., G. L.-C., and D. B. supervision; P. K., G. L.-C., and D. B. conceptualization; P. K., G. L.-C., and D. B. resources; G. L.-C. and D. B. funding acquisition; G. L.-C. and D. B. project administration; D. B. writing-review and editing.

## Footnotes

8 The abbreviations used are:

IL: interleukin
MSU: monosodium urate
CPPD: calcium pyrophosphate dihydrate
LDH: lactate dehydrogenase
LPS: lipopolysaccharide
BMDM: bone marrow–derived macrophage
iBMDM: immortalized mouse bone marrow–derived macrophage
Z-: benzyloxycarbonyl
MDM: monocyte–derived macrophage culture
SASP: secretory phenotype
MEF: mouse embronic fibroblast
DAMP: damage-associated molecular pattern
DMEM: Dulbecco's modified Eagle's medium
FBS: fetal bovine serum
PenStrep: penicillin-streptomycin
PBMC: peripheral blood mononuclear cell
ANOVA: analysis of variance
GSDMD: gasdermin D.
